# Supplementary material for: Addressing disparities in Pre-exposure Prophylaxis (PrEP) access: implementing a community-centered mobile PrEP program in South Florida
Source: BMC Health Serv Res. 2023 Nov 27;23:1311. doi: 10.1186/s12913-023-10277-1 (PMC10683210; doi:10.1186/s12913-023-10277-1)
Supplement: Supplementary file 1 — Supplementary Material 1 [file 12913_2023_10277_MOESM1_ESM.docx]

**Appendix A**

**Key Informant Semi-Structured Interview Guide**

1. Thinking about pre-exposure prophylaxis (PrEP) care, how easy or difficult do you feel it is for clients in this community to get PrEP? Why?
2. What specific barriers to getting PrEP in this community have you heard about? What do you feel are the most important barriers?
3. What do you think would be the most effective way to reach people who may benefit from PrEP in this community? Please be as specific as possible. Are there specific groups within the community who may have different needs?
4. What do you think would make it easier for clients to receive PrEP care (PrEP care includes provider visit, STD screening, laboratory evaluation, prescription)?
5. What do you think would be the most effective place to provide PrEP care for people in this community? Please be as specific as possible, and feel free to propose sites or venues that are not currently available.
6. How acceptable do you feel a mobile clinic setting for PrEP and STD services would be to clients in this community? Why?
7. In selecting a location for positioning a mobile PrEP and STD clinic in this community, what are important considerations? Do you have any specific location in mind that you feel would be good to consider?

**Staff Semi-Structured Interview Guide**

1. How long have you worked with the Mobile PrEP Program?
2. How does Mobile PrEP compare with other ways of obtaining PrEP?
   1. What are some advantages/disadvantages of Mobile PrEP compared with these other programs?
3. In your opinion, how acceptable is the mobile clinic setting and/or venues for clients to receive PrEP and STD services?
   1. Why or why isn’t it acceptable?
4. Thinking about your experience with Mobile PrEP clients, how easy or difficult do you feel it is for them to access our services?
5. Are there specific barriers to accessing Mobile PrEP services?
6. How complicated is Mobile PrEP for:
   1. clients to access?
   2. staff to deliver?
7. To what extent is staff aware of the needs and preferences of the individuals being served by Mobile PrEP?
   1. How "in touch" are staff and leadership with the individuals served by your organization?
8. Have you heard stories about the experiences of clients with Mobile PrEP?
   1. Can you describe a specific story?
9. How well do you think Mobile PrEP meets the needs of the clients we serve?
10. Who are the key influential individuals to get on board with Mobile PrEP?
    1. To what extent will they influence:
       1. others' use of the intervention?
       2. The success of Mobile PrEP implementation in the community?
11. What kinds of changes or alterations do you think you will need to make Mobile PrEP work for different communities/locations?
12. What do you think should NOT be changed?
13. Is there a strong need for Mobile PrEP in the communities we serve?
    1. Why or why not?
14. Do you think community members see a need for Mobile PrEP?
15. What is your perception of the quality of the supporting materials, packaging, and bundling of the Mobile PrEP intervention from the client’s perspective?
16. What supports, such as online resources, toolkits, protocols are available to help you implement Mobile PrEP?
    1. How do you access these materials?
17. Can you describe your working relationships with your colleagues?
18. Can you tell me a story about a time you needed to work with others to solve a problem?
19. Describe activities or initiatives that appear to have highest priority for you within the organization?
    1. What kind of pressure are you feeling to accomplish this?
    2. Where is it coming from? Why?
20. Regarding the Mobile PrEP program, is there anything we haven't touched on during this interview that you'd like to address?
21. What kind of local, state, or national performance measures, policies, regulations, or guidelines influenced the decision to implement Mobile PrEP?
22. How will Mobile PrEP affect your organization's ability to meet these measures, policies, regulations, or guidelines?

**Patient Semi-Structured Interview Guide**

1. How or by whom were you referred to the mobile PrEP clinic?
2. In your opinion, how acceptable was the mobile clinic setting and/or venue to receive PrEP and STD services?
   1. Why or why wasn’t it acceptable?
3. Is there another place or venue where you would prefer to receive services from our program?
4. Thinking about your experience with the mobile PrEP Clinic, how easy or difficult do you feel it was for you to access services? Why?
5. What specific barriers to getting PrEP have you faced or heard about? What do you feel are the most important barriers?
6. What do you feel are some social factors that might stop someone, from your community, from accessing Mobile PrEP services (e.g. stigma or partner disagreement)?
7. What specific tools (e.g. cell phone, direct contact, specific days/times etc.) would help you or people from your community access our program who could benefit from PrEP?
8. Would you recommend Mobile PrEP to other people from your community?
   1. Why or why or why not?
9. Do you have any other suggestions for ways that we can improve our services and/or increase access and acceptability of the mobile PrEP clinic among community members? Please be as specific as possible.
